# Comparison of Physical and Digital Treatment and Documentation of Uncomplicated Cystitis

**DOI:** 10.7759/cureus.17342

**Published:** 2021-08-21

**Authors:** Jonathan Al-Saadi, Max Grönholdt Klein, Jonathan J Ilicki, Therese Djarv

**Affiliations:** 1 Clinical Neuroscience, Karolinska University Hosptal, Stockholm, SWE; 2 Medical Content, Platform24, Stockholm, SWE; 3 Medical Innovation, Platform24, Stockholm, SWE; 4 Emergency Medicine, Karolinska Institute, Stockholm, SWE

**Keywords:** antibiotics, machine learning, mobile medical applications, telemedicine, urinary tract infections, automated patient history collection, ehealth, digital healthcare, cystitis, telehealth

## Abstract

Introduction

Symptomatic criteria have a diagnostic specificity of approximately 90% for uncomplicated cystitis. Today there are triage bots that can collect patient history and document simultaneously. Acute uncomplicated cystitis could potentially be managed digitally, due to the symptom-based approach to diagnosis, but no studies have yet validated this approach.

Aim

We determined the extent of criteria documentation and evaluated adherence to antibiotic recommendations in order to compare physical and digital patient consultations for uncomplicated cystitis.

Materials and methods

This cross-sectional study recruited sixteen 50-year-old women who presented with urinary symptoms to digital healthcare or to three primary physical healthcare facilities. The primary endpoint was the proportion of patients who had two or more documented criteria and received correct antibiotic treatment.

Results

In total, 307 patient visits were included in the study (278 in the digital arm and 40 in the physical arm). The proportion of patients who had two or more documented diagnostic criteria and correct treatment was significantly higher in the digital arm (96 vs 81.6 %, p < 0.001). The total proportion of patients who had fully documented diagnostic criteria did not differ significantly between the arms, however, the proportion with two or more documented criteria was significantly higher in the digital arm (95 vs 77.5%, p < 0.001). The proportion of treated patients who had documented exclusion of diagnostic complicating factors was higher in the digital arm (85.5 vs 0%, p < 0.001).

Conclusions

More patients with urinary tract infection (UTI) now seek digital healthcare providers who have similar or better adherence to antibiotic treatment recommendations and documentation.

## Introduction

Approximately a third of all women have experienced at least one episode of cystitis prior to turning 25 years and nearly half of all women will experience at least one episode of cystitis during their lifetime [1]. Several classification systems of urinary tract infections (UTI) exist. Infectious Diseases Society of America (IDSA) [2] and European Association of Urology (EAU) [3] classify UTI as either uncomplicated or complicated, based on the risk of a complicated course. 

Dysuria, frequency, and urgency are common symptoms of uncomplicated cystitis [4]. In female patients presenting with at least two of the symptoms and absence of vaginal discharge or irritation, the probability of UTI is 90% [4]. Urine dipstick analysis has a sensitivity of 75% and a specificity of 82% [5]. Diagnostic urine tests such as urine dipsticks or cultures are not recommended in otherwise healthy women due to the high positive predictive value of a symptom-based approach [3]. Patients presenting with vaginal discharge or irritation are less likely to have a UTI and more likely to suffer from a vaginal infection or a sexually transmitted disease, which are commonly referred to as diagnostic complicating factors [6]. 

Antimicrobial therapy is recommended for patients with uncomplicated cystitis and moderate to severe symptoms [3], as it has been shown to decrease the duration of symptoms and lead to microbial eradication at the end of treatment compared to placebo [7]. Nitrofurantoin monohydrate/macrocrystals or pivmecillinam are considered as drugs of the first choice by the American [8] and European [3] guidelines.

Recently, telemedicine has emerged as a novel compliment, and in some cases an alternative, to traditional physical consultations [9]. Telemedicine can be defined as the use of medical information that is exchanged from one location to another through technology-enabled devices [10]. Today there are several online symptom checkers and mobile applications which can measure and report physical data [11]. 

The role of digital consultations in today’s healthcare system is disputed and lacks systematic evidence [12]. Studies on critically ill patients have shown that some telemedicine programs can reduce overall mortality, length of hospitalization, and increase rehabilitation [13-16]. Yet, two multicenter randomized placebo-controlled trials found no decrease in hospitalization, morbidity, or mortality when using telehealth to monitor patients with heart failure [17,18]. 

In summary, uncomplicated cystitis is a common diagnosis in pre-menopausal women. Telemedicine has emerged as a novel strategy for primary healthcare practitioners, who aim to diagnose and manage patients in a safe and time-efficient approach, as it allows for remote communication and management of several conditions, such as eczema, prescription renewals, and uncomplicated cystitis. Based on current treatment recommendations, uncomplicated cystitis could potentially be diagnosed and treated through an online doctor consultation [3,6,8]. Unlike the traditional healthcare model, the digital healthcare platform allows for automated documentation, remote consultations, and an algorithmic approach to several diagnoses which possibly could increase adherence to guidelines. However, to the best of our knowledge, no studies have validated this approach and compared it to the traditional physical healthcare delivery model.

Aim

In order to compare physical and digital patient consultations of uncomplicated cystitis, we determined the extent of criteria documentation and evaluated adherence to antibiotic recommendations.

## Materials and methods

Study design

The study consisted of two arms: a digital arm recruited through a digital healthcare provider and a physical arm consisting of patients recruited through three urban primary healthcare facilities outside Stockholm. Data collection in both the physical and digital arms was performed using data from electronic health records (EHR). Patients were provided information on the study and gave their informed consent. 

Study population

Women in the ages 16-50 who presented with symptoms of urinary tract infection [4] were recruited. Both urban and non-urban residents were included. Urban residents were defined as patients living in Stockholm, Gothenburg, or Malmö. Patients were excluded if they were pregnant, over 50 years of age, younger than 16 years of age, or had a known anatomical or functional abnormality within the urinary tract. Anatomical or functional abnormality was defined as a history of polycystic renal disease, nephrolithiasis, neurogenic bladder, diabetes mellitus, immunosuppression, indwelling urinary catheter, or recent urinary tract instrumentation [4]. Symptoms of urinary tract infections were defined as dysuria, frequency, urgency, recognizing symptoms from previous UTI, and lower abdominal pain [4,19].

Outcomes

The primary endpoint was the proportion of patients receiving first-hand antibiotic treatment according to Swedish [20] and EAU guidelines [3] and the proportion of patients with two or more documented diagnostic criteria for UTI. Secondary outcomes were the proportion of patients who were treated and had a documented denial of vaginal discharge or irritation, and subgroup analysis of the primary outcomes.

Data collection

Data was collected from October 2019 - June 2020. Both categorical and numerical data were collected from the EHR. Each visit was counted and analyzed as an individual data point, thus patients presenting again with similar symptoms were included in the analysis. Thus each visit was included in the analysis for patients with multiple visits. Medical health records were accessed after consent was obtained from patients and the healthcare clinic’s operations managers. The ethical permit for this project has been approved by the ethical review board in Stockholm (diary number 2019-03946).

Assessment of the documentation of the diagnostic criteria

Data on documentation was obtained from patients’ medical notes within their EHR. Correct documentation of diagnostic criteria was defined as documenting the presence or lack of urgency, frequency, or dysuria. Furthermore, the physical healthcare clinics had recently incorporated an automated digital self-assessment form which patients filled out in the waiting room before seeing the triaging nurse. We compared the proportion of fully documented criteria before and after the implementation in order to investigate the impact of this self-assessment form.

Assessment of the antibiotic treatment

Data was collected and analyzed from patient’s medical notes within their EHR. Adherence was defined as following STRAMA’s (Swedish Strategic Programme Against Antibiotic Resistance) [20] recommendation with regards to antibiotic type as well as correct dose and duration.

Assessment of the documentation of complicating factors

To investigate documentation of complicating factors [4] that could potentially decrease the probability of a UTI we accessed patients’ medical notes within their EHR and noted if complicating factors (vaginal discharge or vaginal irritation) were documented and negated, documented and present, or not documented at all. 

Statistical analysis

Sample size calculation was based on Kelsey’s formula. It was assumed that 75% of all physical visits fully document the diagnostic criteria of UTI. Allowing for a 25% non-inferiority margin, at least 68 patients in each arm needed to be included in order to have a statistical significance of 0.05 and power of 85%. On the basis of the information that 95% of all physical visits in a similar population obtain correct antibiotic treatment, allowing for a 10% non-inferiority margin, 144 patients in each arm needed to be included in order to maintain the same statistical significance and power.

Statistical analysis was performed with the open-source statistical software R (version 3.6.1, R Foundation for Statistical Computing, Vienna, Austria). Baseline characteristics of the two groups were compared and summarized. All distributed data were tested with the Shapiro-Wilk test to determine whether it was normally distributed or not. Relative risks for incorrect antibiotic treatment and non-complete documentation were calculated and presented as point estimates with 95% confidence intervals. Comparison of the endpoints in the two arms was made through Fisher’s exact test for proportions or Chi-square, depending on sample size.

## Results

Patient recruitment and characteristics

**Figure 1:**
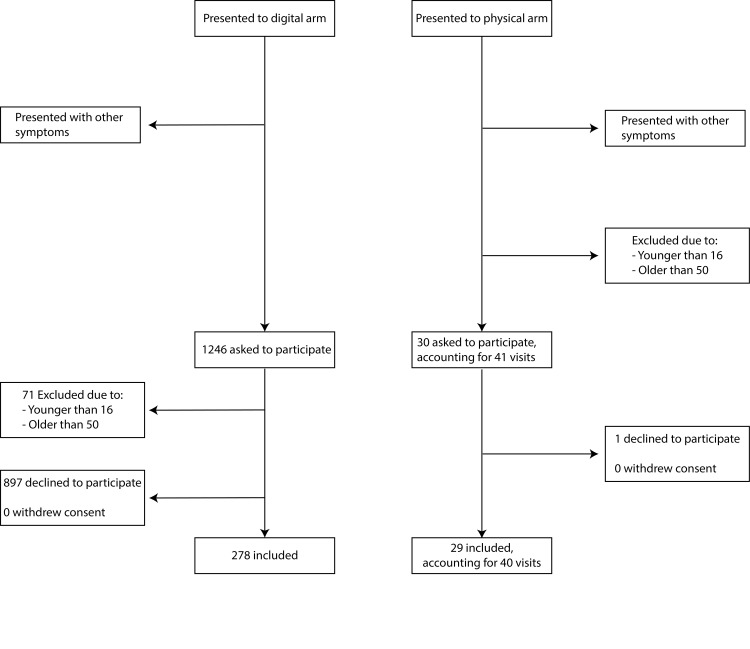
Flowchart for patients included in the study

During the recruitment period (October 2019 to June 2020), a total of 1276 patients were asked to be included. 307 of them consented to be included in the analysis (278 in the digital healthcare arm and 29 in the physical healthcare arm). These patients accounted for a total of 318 visits (278 in the digital arm and 40 in the physical). No patients withdrew consent (Figure 1). In all, 71 patients were excluded in the digital arm due to being older than 50. The baseline demographic characteristics of the patients are shown in Table 1. The mean age of the patients in the digital arm was 32.1 years and 24.4 years in the physical arm. The proportion of patients in the digital arm who were urban residents was 59.0%. Furthermore, 98.9% of the patients had been treated with antibiotics previously in the digital healthcare arm in contrast to 72.4% in the physical arm. 

**Table 1:** Patient characteristics. Continuous variables are presented as the mean with standard deviation between parentheses. Ordinal and categorical variables are presented as numbers and percentages between parentheses.

	Digital	Physical	Overall
Age (SD) - yr	32.1 (9.7)	25.2 (10.4)	31.2 (10.0)
Weight (SD) - kg	70.1 (12.4)	64.9 (12.7)	69.4 (12.5)
Length (SD) - cm	171 (11.2)	167 (9.0)	170 (11.0)
BMI (SD)	24.4 (5.2)	23.5 (5.4)	24.3 (5.25)
Non-urban recidency - no (%)	113 (40.6)	8 (20%)	121 (38.3)
Urban recidency - no (%)	165 (59.4)	32 (80%)	197 (61.9)
Treated - no (%)	173 (62.2)	38 (95)	211 (66.4)
Untreated - no (%)	105 (37.8)	2 (5)	107 (33.6)
Not treated previously - no (%)	3 (1.1)	9 (22.5)	12 (3.8)
Treated previously- no (%)	275 (98.9)	31 (77.5)	306 (96.2)
Symptom duration 1 - 3 days - no (%)	138 (49.6)	26 (65)	164 (51.6)
Symptom duration 4 - 6 days - no (%)	92 (33.1)	6 (15)	98 (30.8)
Symptom duration 7 - 28 days - no (%)	44 (15.8)	8 (20)	52 (16.4)
Symptom duration not documented - no (%)	4 (1.4)	0 (0)	4 (1.3)

Documentation of diagnostic criteria

**Figure 2:**
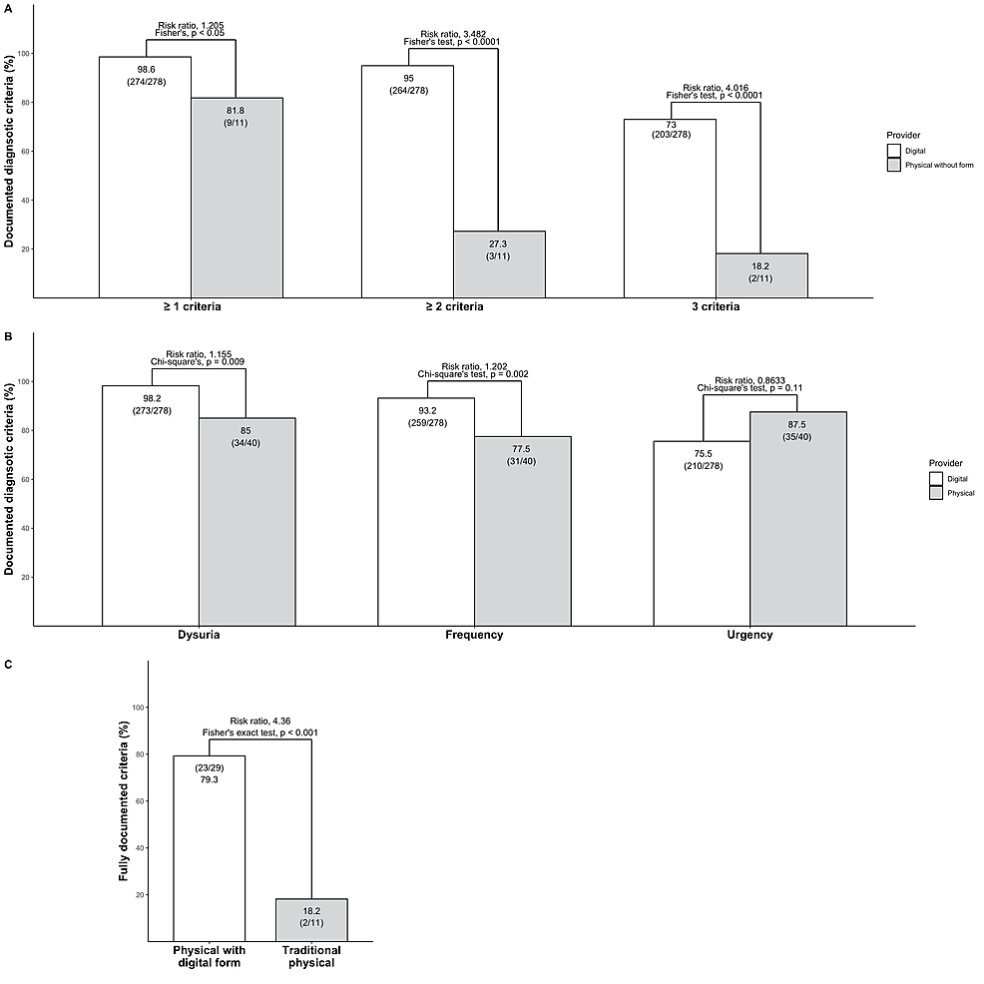
Digital self-assessment increases documentation of diagnostic criteria in both traditional and digital healthcare Multiple bar plots displaying documentation of diagnostic criteria. For categorical variables, Chi-square test or Fisher’s exact test was performed depending on sample size.

The proportion of patients with fully documented diagnostic criteria for UTI was not significantly higher in the digital healthcare arm compared to the physical arm. In the digital arm, 203 out of 278 patients (73%) had fully documented criteria compared to 25 out of 40 (62.5%) in the physical arm (p = 0.02). Patients with ≥ 1 criteria did not differ significantly between the two arms, however, patients with ≥ 2 documented criteria were significantly higher in the digital arm (Figure 2A). Concerning which diagnostic criteria that are documented, no significant difference were found in documenting urgency between both arms (Figure 2B). However, documentation of dysuria and frequency was significantly higher in the digital arm compared to the physical arm. A total of 11 visits to the physical health care centers occurred prior to introducing the digital self-assessment form, and the remaining 29 occurred after. The proportion of patients with fully documented diagnostic criteria before implementation was significantly lower than after implementation (Figure 2C).

Antibiotic treatment

**Figure 3:**
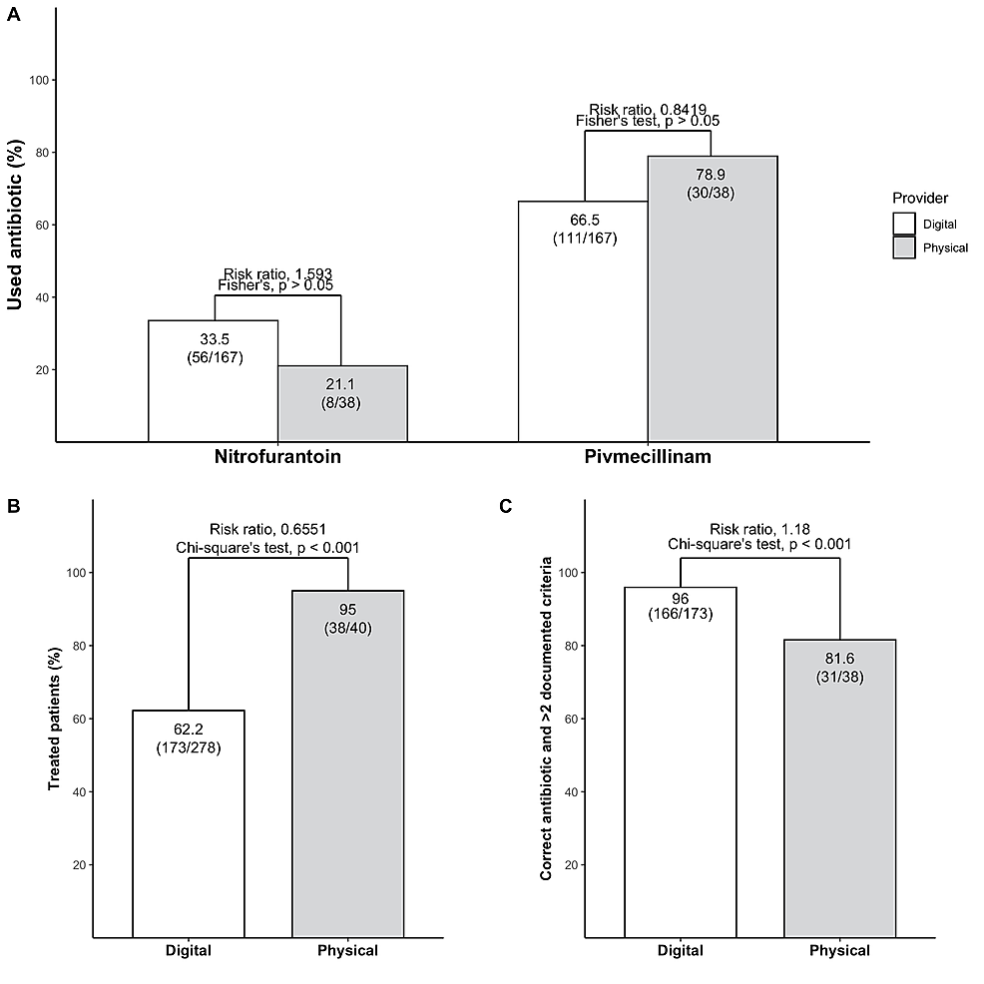
The digital healthcare platform treats fewer patients but documents diagnostic criteria to a greater extent than physical healthcare in treated patients Multiple bar plots displaying treatment of the cohort. Statistical analysis was performed using the Chi-square test.

In both arms, all treated patients received first-hand antibiotics in accordance with STRAMA and EAU guidelines (Figure 3A). The proportion of patients who received pivmecillinam or nitrofurantoin did not differ significantly between both groups. However, the proportion of patients who received antibiotic treatment was significantly higher in the physical arm compared to the digital arm (Figure 3B). In total, 66.4% of the patients received antibiotics, 62.2% in the digital arm, and 95% in the physical arm. The percentage of patients who received correct antibiotic treatment with more than two documented criteria was significantly higher in the digital arm compared to the physical arm (p < 0.001) (Figure 3C). In total, 96% of patients with two or more documented criteria received correct antibiotic treatment in the digital arm compared to 81.6% in the physical arm (p < 0.001).

Untreated patients

**Figure 4:**
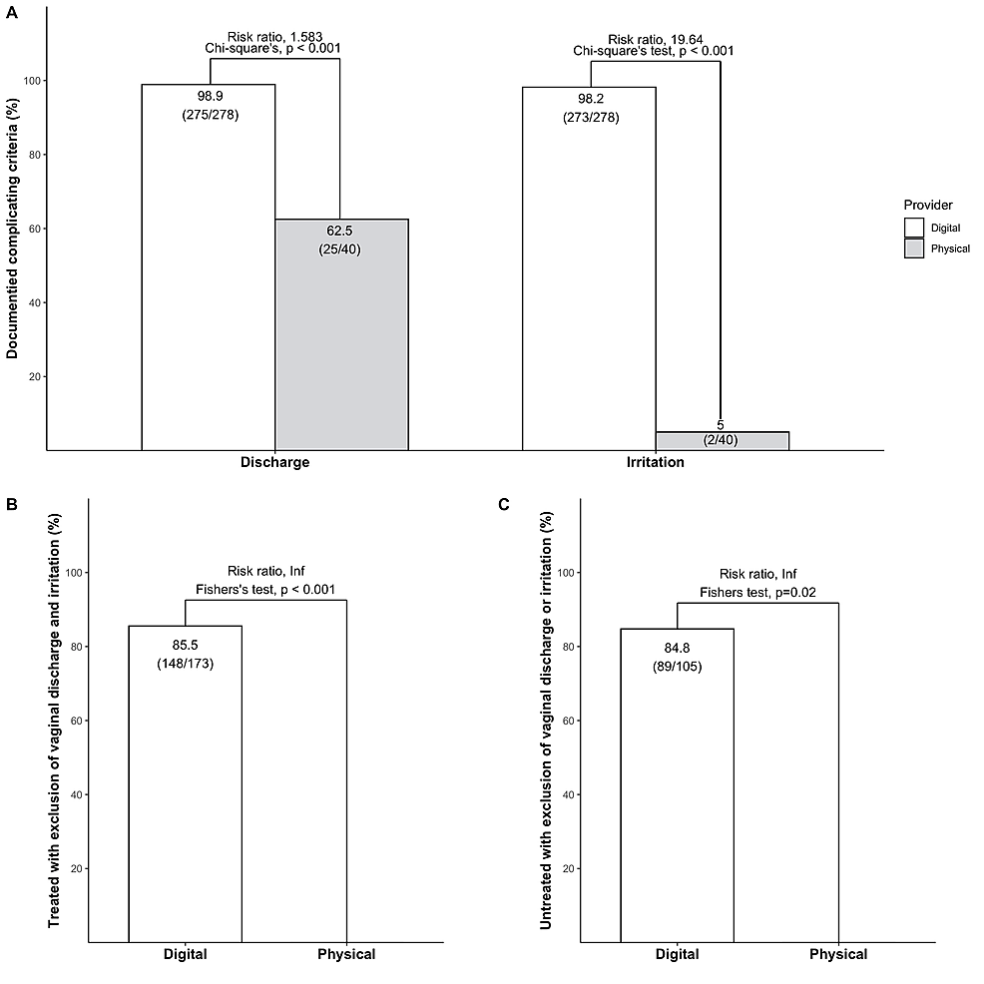
The digital healthcare provider documents complicating factors to a higher extent in treated and untreated patients Multiple bar plots presenting documentation of complicating factors. Statistical analysis was performed with Chi-square test.

Both the proportion of patients with documented presence or lack of vaginal discharge and irritation were significantly higher in the digital arm in comparison to the physical arm (Figure 4A). In total, 84.8% of non-treated patients had a documented exclusion of both vaginal discharge or irritation in the digital arm (Figure 4B). In the physical arm, only one patient was untreated, and in that case, the exclusion of neither vaginal discharge nor irritation was not documented (Figure 4C).

## Discussion

This cross-sectional study is the first to compare physical and digital consultations of uncomplicated cystitis. We determined the extent of adherence to Swedish (STRAMA) [20] and European (EAU) [3] recommendations of antibiotics and documentation of diagnostic criteria. The results of this study suggest that adherence to the correct choice of antibiotics is superior when using digital healthcare compared to traditional physical healthcare. Full documentation, i.e., three documented diagnostic criteria, was not significantly different between the two groups. However, documentation of two or more criteria, which is the number needed for diagnosis, was superior in the digital arm. The proportion of patients with two or more documented diagnostic criteria and subsequent correct antibiotic treatment was significantly higher in the digital arm.

Documentation of diagnostic criteria

Here we showed that a structured but algorithmic approach of a triage bot allows for a consistent negation of symptoms, ensuring safe treatment and importantly only treatment in patients with confirmed uncomplicated cystitis with simultaneous documentation of diagnostic and complicating criteria. 

Antibiotic treatment

Both arms only prescribed pivmecillinam or nitrofurantoin monohydrate/macrocrystals which are the drugs of the first choice according to EAU [3] and STRAMA [20] recommendations. The physical and digital arms both favored pivmecillinam. However, the antibiotic treatment split was more even in the digital arm. The proportion of patients who received antibiotics was significantly lower in the digital arm while the proportion of patients who had documented criteria for the diagnosis of UTI and correct antibiotic treatment was significantly higher. Since we did not perform a longitudinal study it is not possible to conclude why the proportion of treated patients was low. The lower proportion of treated patients could reflect a higher adherence to the symptom-based approach of management. We postulated that patients who presented with complicating factors and thus, not being able to receive treatment with a symptom-based approach due to needing specific physical examinations or diagnostic tests, would be overrepresented in the non-treated group. Indeed, we show here that a high proportion of patients in the digital arm who were untreated had documented signs of vaginal discharge or irritation. Furthermore, it is possible that the higher age and BMI in the digital arm also might lower the proportion of treated patients due to the increased risk of having a more complex disease form [21]. 

Documented complicating factors

It was shown by Bent et al. [4] that a history of vaginal discharge or irritation decreased the specificity of the diagnostic criteria of UTI to almost 50%, rendering the symptom-based approach unreliable. We found that the proportion of patients who had documentation of vaginal irritation or discharge was significantly higher in the digital arm compared to the physical arm, especially in untreated patients. Intriguingly, both arms had a surprisingly high proportion of patients who were treated even though they had a documented presence of vaginal discharge or irritation. This was non-significantly higher in the physical arm. This data is probably an underestimation as physical consultations did not document vaginal irritation. 

Limitations

Unfortunately, this study was underpowered. The low number of patients and only using three clinical healthcare centers in similar geographical locations make the external validity low in the physical arm. At the same time, this made the study feasible in the given timeframe. Moreover, only one digital healthcare provider was used. A major limitation in patient recruitment was the high proportion of patients who declined to participate in the digital arm. Furthermore, this study could only assess documented symptoms and not actual perceived symptoms which might not correlate to the actual perceived symptoms. Finally, digital platforms differ from each other, and it is not clear that these results need be accurate for other digital healthcare providers which do not use the same type of triage bot. 

Future studies

This study reports documentation of diagnostic criteria and treatment at one point in time due to the cross-sectional design of this study. Future studies should consider a longitudinal design to investigate complications and risk for pyelonephritis or re-infections between the two platforms. Furthermore, as the study was underpowered and only conducted in Sweden with three physical healthcare providers in an urban town, more studies are needed to confirm our results with a larger study sample from different locations. 

Interestingly, women in the digital arm had a higher body mass index (BMI) than the physical arm. Higher BMI has been reported to increase the risk of UTI [21]. Moreover, they seemed to be older in general and more women in the digital arm reported that they had been previously diagnosed with a UTI. Perhaps women in the digital arm recognized their symptoms to a greater extent than those in the physical arm, and already knew the management and subsequently chose to seek digital healthcare rather than physical. However, this was not addressed in the scope of this study. Therefore, future studies could explore reasons for why patients choose to seek physical or digital healthcare. In our study, more patients with urinary tract symptoms seemed to seek digital care, reflecting the higher number included in this arm. Although full documentation of all diagnostic criteria was similar in the physical healthcare arm compared to the digital arm, this could be due to the implementation of a digital self-assessment form in the physical arm. Therefore future studies should also focus on comparing traditional physical healthcare platforms with digital platforms. 

## Conclusions

More patients with urinary tract symptoms seek digital healthcare than physical healthcare. The digital healthcare platform was superior compared to the traditional physical healthcare arm in documenting two or more diagnostic criteria for UTI in pre-menopausal women presenting with urinary symptoms.
